# Genetic analysis of *Schistosoma mansoni* in a low-transmission area in Brazil suggests population sharing between wild-hosts and humans and geographical isolation

**DOI:** 10.1371/journal.pntd.0013379

**Published:** 2025-08-11

**Authors:** Karina Varella, Rosana Gentile, Roberto do Val Vilela, Silvana Carvalho Thiengo, Aline dos Santos Moreira, José Roberto Machado-Silva, Thiago dos Santos Cardoso, Sócrates Fraga da Costa-Neto, Beatriz de Lima Alessio Müller, Alexandre Araujo Cunha dos Santos, Arnaldo Maldonado Junior

**Affiliations:** 1 Programa de Pós-Graduação em Biologia Parasitária (PPGBP), Instituto Oswaldo Cruz, Fundação Oswaldo Cruz, Manguinhos, Rio de Janeiro, Brazil; 2 Laboratório de Biologia e Parasitologia de Mamíferos Silvestres Reservatórios, Instituto Oswaldo Cruz, Fundação Oswaldo Cruz, Manguinhos, Rio de Janeiro, Brazil; 3 Laboratório de Referência Nacional para Esquistossomose – Malacologia, Instituto Oswaldo Cruz, Fundação Oswaldo Cruz, Manguinhos, Rio de Janeiro, Brazil; 4 Plataforma de Sequenciamento de DNA por eletroforese capilar (Sanger – RPT01A), Plataforma de Análise de Fragmentos de DNA e Microssatélites (RPT01D), Laboratório de Genômica Aplicada e Bioinovações, Instituto Oswaldo Cruz, Fundação Oswaldo Cruz, Manguinhos, Rio de Janeiro, Brazil; 5 Laboratório de Helmintologia Romero Lascasas Porto, Departamento de Microbiologia, Imunologia e Parasitologia, Faculdade de Ciências Médicas, Universidade do Estado do Rio de Janeiro, Vila Isabel, Rio de Janeiro, Brazil; 6 Campus Fiocruz Mata Atlântica CFMA, Fundação Oswaldo Cruz, Rio de Janeiro, Brazil; Zhejiang Wanli University, CHINA

## Abstract

**Background:**

The fluke *Schistosoma mansoni* is the causative agent of intestinal schistosomiasis, a neglected tropical disease, and remains prevalent in certain regions of Brazil. In the municipality of Sumidouro, state of Rio de Janeiro, Brazil, a low-endemic area for *S. mansoni*, water rats (*Nectomys squamipes*) are naturally infected by this trematode. The *S. mansoni* populations infecting humans and water-rats in Sumidouro exhibit distinct patterns of cercarial emergence (chronotypes) and phenotypic differences between hosts. Previous studies have shown that the adaptation of *S. mansoni* populations to human hosts (diurnal chronotype) and water rats (nocturnal chronotype) could result in prezygotic isolation. To test this hypothesis, we employed the mitochondrial cytochrome c oxidase subunit 1 gene (MT-CO1) and microsatellite loci as genetic markers.

**Principal findings:**

We assessed the population structure between the definitive host species and geographically distant isolates collected from two endemic localities (Pamparrão–PAM and Encanto-Soledade–ENC-SOL) in Sumidouro. Additionally, we evaluated the phylogenetic relationships between *S. mansoni* from Sumidouro and those from other countries. Five haplotypes of the MT-CO1 gene were identified, with haplotypes 3 and 4 exclusive to ENC-SOL, and haplotypes 1, 2, and 3 were shared between humans and water rats. Haplotype 1 was also shared with other Brazilian localities, South American countries and a single locality in West Africa. The remaining haplotypes were exclusive to Sumidouro, indicating local genetic diversity. Population structure analysis revealed no genetic differentiation associated with host species but rather geographical structuring, probably due to the sedentary habits of rodents and the limited movement of humans between localities. This finding indicates that *S. mansoni* populations with different chronotypes are not genetically isolated and that significant gene flow occurs between them.

**Conclusions:**

In conclusion, our findings confirm that wild rodents contribute to the maintenance of the *S. mansoni* life cycle in Sumidouro and can serve as indicators of local transmission hotspots.

## Author summary

The parasite *Schistosoma mansoni* causes intestinal schistosomiasis in humans, resulting in abdominal pain, diarrhea, and blood in the stool. In the municipality of Sumidouro, state of Rio de Janeiro, Brazil, in addition to human transmission, we found wild rodents infected by *S. mansoni*. In this work, we described and compared the genetic diversity of *S. mansoni* isolates from wild rodents and humans at two localities in Sumidouro. We did not observe genetic differences between *S. mansoni* isolates from rodents and humans, indicating the sharing of the parasite. However, we observed two genetic clusters corresponding to the two localities. These results reveal the importance of wild rodents in maintaining the parasite cycle in the Sumidouro region, which contributes to transmission in humans.

## Introduction

Schistosomiasis is a neglected tropical disease caused by digenetic trematodes of the genus *Schistosoma* Weiland, 1858 [1,2]. According to the World Health Organization (WHO) [3], schistosomiasis is transmitted in more than 78 countries, predominantly in tropical and subtropical regions with low socioeconomic status, inadequate access to safe water, and poor sanitation. This disease is responsible for approximately 280,000–500,000 thousand deaths annually [2] and poses a significant global health burden, with an estimated 1.75 million disability-adjusted life years (DALYs) [4].

The WHO has set a target to eliminate schistosomiasis as a public health problem by 2030 through a multifaceted approach. This includes preventive chemotherapy for risk groups, improved access to WASH (safe water, sanitation, and hygiene) and education, environmental management, and snail control. However, the transmission dynamics, disease pathology, and effectiveness of control strategies vary by geographic region and are influenced by factors such as environmental conditions, intermediate host habitat, age at infection and treatment, exposure patterns and presence of reservoir hosts [3].

In the Americas, *Schistosoma mansoni* Sambon, 1907, is the species responsible for intestinal schistosomiasis, with 10 countries and territories considered endemic. In northeastern Brazil and central Venezuela, approximately 25 million people are at risk [5]. Recent studies have provided evidence of the zoonotic nature of *S. mansoni* in West Africa [6,7], where parasite lineages are shared between humans and wild rodent reservoirs at transmission sites, potentially contributing to human reinfection following therapeutic treatment [7].

In Brazil, some wild rodent species, particularly those in the genera *Nectomys* Peters, 1861, and *Holochilus* Brandt, 1835, have been identified as natural reservoirs of *S. mansoni* [8–10]. These semi-aquatic rodents are highly prone to *S. mansoni* infection because of their habitat preferences [11–13]. The water rat *Nectomys squamipes* Brants, 1827, is of particular interest, as it has been found to be naturally infected with *S. mansoni*. This rodent has crepuscular or nocturnal habits and occurs near streams, rivers, and flooded areas [11,14].

The municipality of Sumidouro is a low-endemic area for *S. mansoni*, with a prevalence in humans that is close to 10%, where the rural population remains infected despite regular treatment efforts [9,13]. In this region, *N. squamipes* populations present high prevalence rates (≅ 30%) of *S. mansoni* infection across different localities [13,15–18]. These localities include small rural areas where water bodies are used for agriculture, consumption, and leisure, creating a conducive environment for transmission [13].

Schistosomiasis surveys in Sumidouro date back to 1959, with research intensifying in the 1980s to investigate the epidemiological importance of water rat populations [13,19]. Since the 1990s, multidisciplinary studies have been conducted to understand the role of *N. squamipes* in schistosomiasis transmission dynamics [9,13], as well as the population biology of the intermediate host snail *Biomphalaria glabrata* (Say, 1818) [20] and health education and treatment of the human population [21,22].

*Nectomys squamipes* often inhabits areas close to human dwellings [23,24], is highly susceptible to *S. mansoni* infection [12,15,24], and can shed viable eggs throughout its lifespan by infecting *B. glabrata* [8,25]. These facts contribute for maintaining high infection rates, which are often higher than those of the human population that continues to harbor the parasite [13]. *Nectomys squamipes* populations do not appear to suffer significant fitness costs from infection, as reproductive females are infected, and individuals exhibit high longevity with no observable impact on recruitment or survival rates due to *S. mansoni* infection [12]. Furthermore, the low occurrence of granulomatous inflammatory processes in infected *N. squamipes* is associated with hepatic lipid accumulation, which may serve as a protective mechanism against the parasite [10]. These findings highlight the role of *N. squamipes* as a wild reservoir for *S. mansoni*, contributing to the persistence of the parasite in endemic areas and facilitating transmission [12,23,24].

Chronobiological studies have shown that the cercariae emergence rhythms of *S. mansoni* isolates in Sumidouro are synchronized with the activity patterns of their definitive hosts [26]. Laboratory experiments with *B. glabrata* snails exposed to *S. mansoni* miracidia from naturally infected *N. squamipes* and humans revealed a diurnal pattern (diurnal chronotype) in human isolates and a crepuscular/nocturnal pattern (nocturnal chronotype) in rodent isolates [26]. Freire et al. [27] further observed biological differences between *S. mansoni* isolates from humans and rodents through experimental infection in snails, with rodent isolates showing a shorter pre-cercarial period and greater infectivity, suggesting the existence of host-specific populations.

Morphological and morphometric studies have also revealed significant phenotypic differences between adult *S. mansoni* from *N. squamipes* and those from human isolates in Sumidouro [28,29]. These findings suggest that the presence of different hosts creates environmental heterogeneity, which favors the emergence of new *S. mansoni* phenotypes [30].

Some researchers have proposed that the adaptation of *S. mansoni* populations to different hosts could lead to prezygotic isolation [31,32]. Based on the distinct chronotypes [26] and phenotypic differences [28,29] observed in *S. mansoni* populations infecting humans and water rats in Sumidouro, we tested the hypothesis of prezygotic isolation using the mitochondrial cytochrome c oxidase subunit 1 (MT-CO1) gene and microsatellite loci as genetic markers.

In this study, we investigated the genetic profile of *S. mansoni* populations from Sumidouro by considering (i) the phylogenetic relationships between *S. mansoni* in Sumidouro and other countries, (ii) the spatial distance between isolates, (iii) temporal variation up to 22 years of difference between samples, and (iv) representativeness of the genetic sequence data from parasite eggs compared with adult schistosomes. Given the importance of *N. squamipes* as a reservoir, this research will contribute to a deeper understanding of the complex epidemiology of schistosomiasis in a multi-host context and inform the development of targeted control strategies in areas where water rats are present.

## Materials and methods

### Ethics statement

Authorization to obtain epidemiological data and collect human biological material was granted under number CAAE: 22322619.3.0000.5248, by the Research Ethics Committee of the Oswaldo Cruz Institute–CEP/Fiocruz. The euthanasia of the rodents followed the guidelines of the Animal Care and Use Committee of the American Society of Mammalogists [33] and the Brazilian Guide to Good Practices for Euthanasia in Animals by the Ethics, Bioethics, and Animal Welfare Committee of Brazil’s Federal Council of Veterinary Medicine [34]. Permits for rodent capture and handling were issued by the Chico Mendes Institute for Biodiversity Conservation (ICMBio/SISBIO, license numbers 13373–1, 45839–1, and 63023–4); procedures were approved by the ethics committees on animal use of the Oswaldo Cruz Foundation and the Oswaldo Cruz Institute (CEUA-FIOCRUZ, license number LW-39/14; and CEUA-IOC, license number L-036/2018).

### Study area

**Fig 1:**
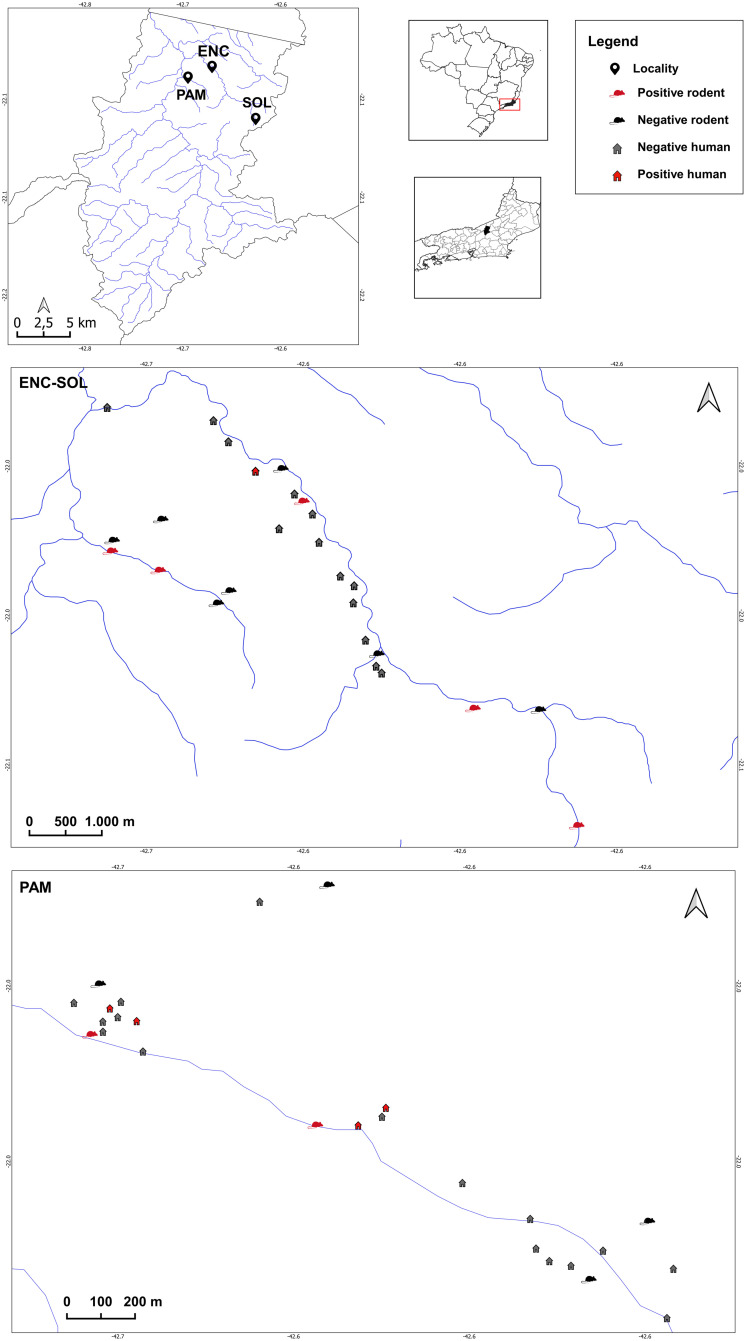
Sampling localities of *Schistosoma mansoni* populations from humans and from the water rat *Nectomys squamipes* in the ENC-SOL (Encanto and Soledade) and PAM (Pamparrão) localities in Sumidouro, state of Rio de Janeiro, Brazil. The symbols represent the sampling sites, and the colors represent positive (red) and negative (green) hosts (humans or *N. squamipes*) for *Schistosoma mansoni*. Software: QGIS 3.22. Source: Instituto Brasileiro de Geografia e Estatística (IBGE). Continuous cartographic base (2020). Available from: https://www.ibge.gov.br/geociencias/downloads-geociencias.html?caminho=cartas_e_mapas/bases_cartograficas_continuas/bc250/versao2023/; https://www.ibge.gov.br/geociencias/downloads-geociencias.html?caminho=cartas_e_mapas/bases_cartograficas_continuas/bc25/rj/versao2018_edgv_3.0/.

This study was conducted in three localities within the municipality of Sumidouro (22º02’59“S, 42º40’29”W) situated at an altitude of 355 meters, in the state of Rio de Janeiro, Brazil. Sumidouro is an endemic area for *S. mansoni* located in the Centro Fluminense mesoregion. The specific study sites included I - Encanto (22°02’37.5”S, 42°37’13.3”W), II - Soledade (22°3’10”S, 42°35’58”W) and III - Pamparrão (22°02’02.5”S, 42°38’59.1”W) (Fig 1). The local population is 15,206 inhabitants [35], and the municipality covers an area of 413,407 km². Sumidouro is located within the Piabanha Hydrographic Basin, which is part of Hydrographic Region IV and spans 4,484 km². The Piabanha Basin, along with the Paquequer and Preto sub-basins, are among the major sub-basins forming the Paraíba do Sul River.

In the localities of Encanto/Soledade (ENC-SOL), human stools and rodent samples were collected from 2001 to 2003 and in 2022 and 2023. In Pamparrão (PAM), human samples were collected in 2019. Only one stool sample was collected from each resident per study locality and period. Rodent samples were collected in 2021 and 2022. ENC-SOL was treated as a single locality in all analyses because they are part of the same water basin and drained by the same river, therefore, in the context of disease transmission, they cannot be considered separately. Only one specimen of *N. squamipes* positive for *S. mansoni* was collected in the Santa Cecília locality (22°01’14.3“S, 42°39’24.3”W) in Sumidouro, thus it was included only in the phylogenetic analyses.

### Sampling methods of *S. mansoni* populations

Human stool samples were collected from residents in each locality who voluntarily participated in the research. All individuals over 18 years of age who agreed to participate by signing an informed consent form were included in the study. For those under 18 years of age, consent was obtained from parents or legal guardians by signing an informed consent form. Stool samples were collected in labeled plastic containers provided to the participants. The stool samples were analyzed using the Kato-Katz method [36], standard method recommended by WHO [3], with three slides prepared for each stool sample, as well as the Lutz method [37], with three slides per sample. The same human stool samples were subjected to both coprological methods to increase the sensitivity of the diagnosis since the studied localities are of low endemicity. The Kato-Katz method was used to quantify eggs only in human stools. All coprological examinations were performed on the day of collection. Subsequently, the sediment from the Lutz method of the positive samples was examined under a stereoscopic microscope to isolate individual parasite eggs, which were then stored in 0.85% saline solution at -20°C for molecular analyses.

To sample rodents, we established 150-meter linear transects with six transects in Pamparrão and five transects in Encanto and Soledade. Each transect contained 15 capture point stations 10 m apart. Tomahawk Live Traps (Model 201 traps 16” × 5” × 5”, Hazelhurst, Wisconsin), baited with a mixture of peanut butter, banana, oats, and bacon, were placed on the ground, along streams and flooded areas, the natural habitat of *N. squamipes* [11]. For a comprehensive overview of the rodent study conducted from 2001 to 2006, refer to Gentile et al. [24].

*Nectomys squamipes* specimens captured were identified by their external morphology and transported to a field laboratory. Subsequently, the rodents were euthanized using a lethal dose of anesthetic and subjected to perfusion of the portal-hepatic system, according to Smithers and Terry [38], to recover adult *S. mansoni* in the mesenteric and portal veins. The anesthetic protocols included ketamine (100 mg/mL) combined with acepromazine (10 mg/mL) at a ratio of 9:1 (dose of 0.15 mL/100 g body weight). The perfusion pressure was provided by a rotary peristaltic pump with a foot-operated switch. Following euthanasia, the abdominal and thoracic cavities of the rodents were opened, and the rib cage was removed. Next, the hepatic portal vein was incised, the perfusing needle was positioned in the left ventricle of the heart, and the pump was initiated. After all procedures, the rodents were taxidermized. Biosafety protocols and personal safety equipment were utilized during all procedures involving animal handling and biological sampling. All *N. squamipes* specimens were deposited at the Integrated Collection of Wild Reservoir Mammals (COLMASTO), of the Laboratory of Biology and Parasitology of Wild Reservoir Mammals (LABPMR), formally the Laboratory of Biology and Control of Schistosomiasis (LBCE).

Positive cases were confirmed by the presence of adult *S. mansoni* after the perfusion technique. The recovered adult schistosomes were washed in 0.85% saline solution to remove tissue debris, counted and sexed under a stereoscopic microscope, and stored in 70% ethanol solution for molecular analyses. The stools of positive rodents for *S. mansoni* were subjected to coprological examinations (Lutz method). The sediment of the positive samples was examined under a stereoscopic microscope to isolate parasite eggs individually with the aid of a micropipette. All coprological examinations were performed on the day of collection. Each isolated egg was placed in a Petri dish and subjected to successive washings in 0.85% saline solution to remove fecal debris and stored in the same solution at -20°C for molecular analyses. Before being subjected to molecular techniques, each egg was placed in a Petri dish and washed in autoclaved distilled water.

### Genomic DNA isolation

We isolated genomic DNA from *S. mansoni* using the QIAamp DNA Mini Kit (QIAGEN, Hilden, Germany) according to the manufacturer’s instructions for adult schistosomes and according to Webster et al. [39] for eggs. We used only adult schistosomes obtained from rodents and eggs obtained from humans and rodents. Before DNA isolation, each adult schistosome and egg were washed in autoclaved distilled water to remove residual ethanol or saline solution. The eggs were placed in a Petri dish with the aid of a micropipette and subjected to successive washings with autoclaved distilled water.

### MT-CO1 analyses

#### Amplification and sequencing.

Mitochondrial cytochrome c oxidase subunit I gene (MT-CO1) partial region, with ≅ 600 bp, was amplified from 146 *S. mansoni* specimens, including eggs and adult schistosomes from 65 rodents, and 82 *S. mansoni* eggs from 20 human feces samples. Amplifications of the MT-CO1 by polymerase chain reaction (PCR) were performed individually for each adult schistosome and egg using the primer pair described by Morgan et al. [40], NHMMOR F (GCT TAG GTA GAG TAG TAT GGG GTC) and NHMMOR R (CTT AGC CAC CCA CAA CTT AG). Amplifications were performed using T100 PCR thermal cycler in a 96-well plate (Bio-Rad, Hercules, USA). Each reaction contained 12.5 μL of PCR Master Mix, 2X (Promega Corporation) (50 units/mL Taq DNA polymerase, 400 μM dATP, 400 μM dGTP, 400 μM dCTP, 400 μM dTTP, 3 mM MgCl2), 0.5 μL of each primer (10 μM), and 1 μL of genomic DNA (≅ 10–20 ng), and ultrapure water in a total volume of 25 μL. The thermal cycling conditions were 95°C for 2 min; 40 cycles at 95°C for 30 s, 55°C for 30 s, and 72°C for 1 min; and a final extension at 72°C for 10 min [41]. Successfully amplified amplicons were purified using the Illustra GFX PCR DNA and Gel Band Purification Kit (GE Health care, Little Chalfont, Bucks, UK), following the manufacturer’s protocol, and cycle sequenced using the Big Dye Terminator v3.1 Cycle Sequencing Kit (Applied Biosystems, Carlsbad, California, USA). Cycle-sequenced product precipitation, formamide resuspension, and sequencing were conducted on the Fiocruz capillary sequencing (SANGER) platform P01-001A-RPT/FIOCRUZ (https://plataformas.fiocruz.br/). Sequencing was performed using an ABI 3730xl DNA Analyzer (Applied Biosystems).

#### Molecular phylogenetic and phylogeographic analyses.

The resulting DNA sequencing reads were assembled into contigs and edited for ambiguities using Geneious version 2023.0.3 [42], resulting in consensus sequences. All haplotype sequences were deposited in GenBank (accession numbers PQ738167-PQ738171).

Three MT-CO1 datasets were used in this study: (1) for the phylogenetic analysis, we used unique sequences obtained in the present study and others deposited in Genbank representing Sumidouro haplotypes, and sequences deposited in Genbank representing across the geographic distribution of *S. mansoni*, with *Schistosoma rodhaini* (accession numbers: AY446142 and AY446143) as an outgroup (S1 Table); (2) for genetic structure analysis, we used sequences of *S. mansoni* obtained in the present study from two studied localities (PAM and ENC-SOL); and (3) for temporal analysis (22 years apart), we used sequences obtained in the present study from the ENC-SOL locality.

For each dataset, MT-CO1 sequences were aligned using the MUSCLE algorithm [43] through Geneious. The resulting alignments were manually trimmed of poorly aligned extremities using Mesquite, version 3.81 [44]. Substitution saturation was assessed via the Xia test [45] using DAMBE, version 6.4.79 [46].

Phylogenetic analyses were performed only for the dataset that included our five haplotype sequences of *S. mansoni* from Sumidouro and other sequences throughout its geographic distribution on different continents and outgroups that are deposited in Genbank. Phylogenetic reconstructions using maximum likelihood (ML) as the optimality criterion were generated using the IQ-TREE, version 2.3.6 online web server [47]. Evolutionary model selection was implemented with ModelFinder [48] in IQ-TREE using the Bayesian information criterion (BIC). Node support was assessed by the SH-like approximate likelihood ratio test (SH-aLRT) [49] and ultrafast bootstrap (UFBoot) [50] after 1,000 replicates.

Bayesian inference (BI) phylogenetic analyses were performed using MrBayes, version 3.2.6 [51], executed on XSEDE through the CIPRES Science Gateway [52]. Independent GTR + I + G (general time-reversible nucleotide substitution model, with a proportion of invariable sites and a gamma distribution of rates among sites) models were used for each codon position, with unlinking of base frequencies and parameters. Markov chain Monte Carlo (MCMC) sampling was performed for 10,000,000 generations with four simultaneous chains in two runs. Node support was assessed by Bayesian posterior probabilities (BPP), which were calculated from trees sampled every 100 generations, after removing the first 25% ‘burn-in’ generations. Sampling adequacy was assessed using the program Tracer, version 1.7.2 [53], to calculate the effective sample sizes (ESSs) of the parameters. Values above 200 effectively independent samples were considered robust.

Haplotype networks were inferred only for *S. mansoni* sequences from Sumidouro using the program PopART, version 1.7 [54], under the median-joining method (Bandelt et al., 1999). We used DNAsp, version 5.10.1 [55], to organize *S. mansoni* sequences from Sumidouro into groups according to geographic locality. Additionally, using DNAsp, the genetic diversity of each group was calculated in terms of the number of haplotypes (H), number of polymorphic sites (S), haplotype diversity (Hd), and nucleotide diversity (π).

#### Genetic structure analyses.

Analysis of molecular variance (AMOVA) [56] and the fixation index (*F*_ST_) [57] were calculated only for *S. mansoni* sequences from Sumidouro using the Arlequin software package, version 3.5.2.2 [58]. We used AMOVA to analyze genetic variability between and within previously defined groups and *F*_ST_ to measure levels of genetic differentiation between groups (between hosts, between localities and separately, considering hosts by localities). We also used the program Arlequin to assess deviation from neutrality through Tajima’s D [59] and Fu’s Fs [60] tests for each of the previously determined groups.

#### Discriminant analysis of principal components.

Haplotype variations between different groups (between hosts, between localities and separately, considering hosts by localities) were analyzed only for *S. mansoni* sequences from Sumidouro using discriminant analysis of principal components (DAPC), as exemplified in Varella et al. [61]. This analysis makes it possible to investigate variations between groups and select principal components (PCs) to explain the existing variation in the dataset [62,63]. The number of PCs to be used in the analysis was determined by a cross-validation optimization procedure [63]. The percentage of correctly classified haplotypes for their original group was also determined, and differences between groups were tested using Wilk’s Lambda statistics. DAPC was performed using the adegenet package [64], and Wilk’s lambda statistics were performed using the rrcov package [65] in the R software environment, version 4.1.3 [66].

### Microsatellite analyses

#### Amplification and genotyping.

Seven microsatellite loci were amplified from 124 *S. mansoni* specimens, including eggs and adult schistosomes from 21 rodents, and 34 *S. mansoni* eggs from eight human feces samples. The amplification of the microsatellite alleles of seven loci via PCR was performed individually for each adult schistosome and egg of *S. mansoni* from Sumidouro using the primer pairs described in Table 1. Amplifications were performed using a T100 PCR thermal cycler in a 96-well plate (Bio-Rad, Hercules, USA).

**Table 1:** Characteristics of the microsatellite loci used in this study.

Multiplex	Locus	Motif	Foward primer	Reverse primer	Dye	Size range	Reference
GROUP I	**SMMS 3**	(TAA)_12_	GGTCAACAGCAATATCAGC	GATCATCTTCATGACGTCG	NED	176–209	Silva et al. [77]
	**SMMS 16**	(TTA)_11_	CACCCATTGTCTTAAAACC	GATGTCACACCCTC	VIC	211–229	Silva et al. [77]
	**SMMS 18**	(AAT)_13_	CACCTCAACACCTATG	GTTGGAAACACATTGGGC	PET	195–228	Silva et al. [77]
GROUP II	**15J15A**	(ACT)_7_	TGTGGTTAATCGCTGCTACC	GTTTCATGCCAACTGCGTCTC	6-FAM	208–232	Blanton et al. [78]
	**29E6A**	(ATC)_7_	ACATCCAGCTGACGAGTCC	ACTGCCCTATTCCTAACTGGC	VIC	160–178	Blanton et al. [78]
	**SM13–478**	(AAT)_10_	CAGGAATTTGTATTGTTCTGCTGTC	ACAGTGGCTAACTGACTACG	PET	225–258	Blanton et al. [78]
	**1F8A**	(ATT)_6_	GGCTTCAGTCGTCGTGTTC	GCTTCTTCGTTGCCACACTC	6-FAM	151 - 172	Blank et al. [79]

The PCRs were standardized at the Fiocruz Fragment Analysis Platform, P01-001D-RPT/FIOCRUZ (https://plataformas.fiocruz.br/), and performed in two multiplex groups. For each multiplex, the forward primers were fluorescently labeled with 6-FAM, VIC, NED, and PET dyes (Applied Biosystems, Foster City, USA) (Table 1).

Each reaction contained 25 μL of PCR Master Mix, 2X (Promega Corporation) (50 units/mL Taq DNA polymerase, 400 μM dATP, 400 μM dGTP, 400 μM dCTP, 400 μM dTTP, 3 mM MgCl2), 1 μL of each primer (10 μM), and 2 μL of genomic DNA (≅ 10–20 ng), and ultrapure water in a total volume of 50 μL. The thermal cycling conditions were 95°C for 2 min; 35 cycles at 95°C for 30 s, 50°C for 30 s, and 72°C for 30 s; and a final extension at 72°C for 5 min.

All PCR multiplex products were run with a standard size of GS500 LIZ on an ABI 3130xl Genetic Analyzer at the Fragment Analysis Platform, P01-001D-RPT/FIOCRUZ. Allele fragment lengths were quantified using the GeneMapper software, version 4.1 (Applied Biosystems).

Micro-Checker software, version 2.2.3 [67], was used to verify scoring errors, large allele dropout and the presence of null alleles. The presence of linkage disequilibrium was verified by a likelihood ratio test [68] that was performed in each group using Arlequin software package.

#### Genetic diversity analyses.

The number of individuals in each group and the number of alleles per locus of each group were calculated using GenAIEx, version 6.503 [69]. The observed (*H*_*O*_) and expected (*H*_*E*_) heterozygosities were calculated using Arlequin. The inbreeding coefficient (*F*_*IS*_) was generated using FSTAT 2.9.4 [70]. Deviations from Hardy–Weinberg equilibrium per locus for each group were estimated with GenAIEx.

#### Genetic structure analyses.

Three SSR datasets were used in this study: (1) for temporal analysis evaluation (22 years apart), we used microsatellite loci amplified of *S. mansoni* from the ENC-SOL locality between 2001–2003 and 2019–2023 periods; (2) for genetic structure analysis between definitive host species (humans and *N. squamipes* isolates), we used microsatellite loci amplified of *S. mansoni* from the two studied localities (PAM and ENC-SOL) separately; (3) for genetic structure analysis between localities (ENC-SOL and PAM), we used microsatellite loci amplified of *S. mansoni* using only the 2019–2023 period considering the two definitive hosts together (humans and *N. squamipes*).

We used AMOVA to analyze genetic variability between and within previously defined groups and *F*_ST_ to measure levels of genetic differentiation between groups for three datasets. AMOVA and *F*_ST_ were calculated using Arlequin. We examined the patterns of connectivity among *S. mansoni* localities (PAM and ENC-SOL) through network analysis, which was based on *F*_ST_ values [71] without a priori premise of genetic groups, using EDENetworks, version 2.18 [72]. The nodes on the network corresponded to the populations, and the threshold was the maximum distance considered as a link in the network. All links with distances above this threshold value were removed. The number of edges connected to a node, i.e., the degree of connectivity, indicates how strongly a population is connected to other populations. In contrast, the value of the shortest paths between other nodes passing through a node was defined as the betweenness centrality.

The program Structure, version 2.3.4 [73], which implements a Bayesian model-based clustering method for inferring population structure, was used to identify distinct genetic populations and to assign individuals to populations. We evaluated whether the genetic clusters (K) corresponded to the definitive hosts (human or water rat) or to the localities studied. We performed the runs for K = 1–10 with 10 replicates, each with 10^6^ iterations by Markov Chain Monte Carlo (MCMC–2.5 × 10^5^ burn-in), using correlated allele frequencies and assuming the admixture model of ancestry. To find the optimal K, we used Structure Selector, which applies the ΔK method from Evanno et al. [74]. The consensus matrix Q was generated from ten optimal K replicates and visualized in Distruct, version 1.1 [75]. Major and minor modes of clustering patterns at each K were determined using Clumpak [76].

## Results

### MT-CO1 analyses

#### Phylogenetic analysis—Sumidouro and other endemic areas of the world.

The dataset for phylogenetic analyses comprised 124 sequences of the MT-CO1 gene (555 bp), 122 of which refer to *S. mansoni* from Brazil and other countries, as well as two sequences from *S. rodhaini* as an outgroup. The localities were from Africa, represented by Kenya, Tanzania, Uganda, Zambia, Cameroon, Nigeria, Ghana, Liberia, Mali, and Senegal; the Americas, represented by Brazil, Guadeloupe, Puerto Rico and Venezuela; and Madagascar, Oman and Egypt. Xia’s test provided no evidence for substitution saturation in the data matrix for phylogenetic analyses.

The ML best-fit model chosen by ModelFinder in IQ-TREE under the BIC was TPM3 + F + G4, with four substitution rate categories and a gamma shape parameter of α = 0.1404, resulting in an ML phylogenetic tree with a lnL = -2076.7829 score. The BI MCMC sampling, after 25% ‘burn-in’, resulted in a mean estimated marginal likelihood of phylogenetic trees = –2112.8715 (standard deviation = 12.8003; median = −2112.516). The ESSs were robust for all the parameters.

**Fig 2:**
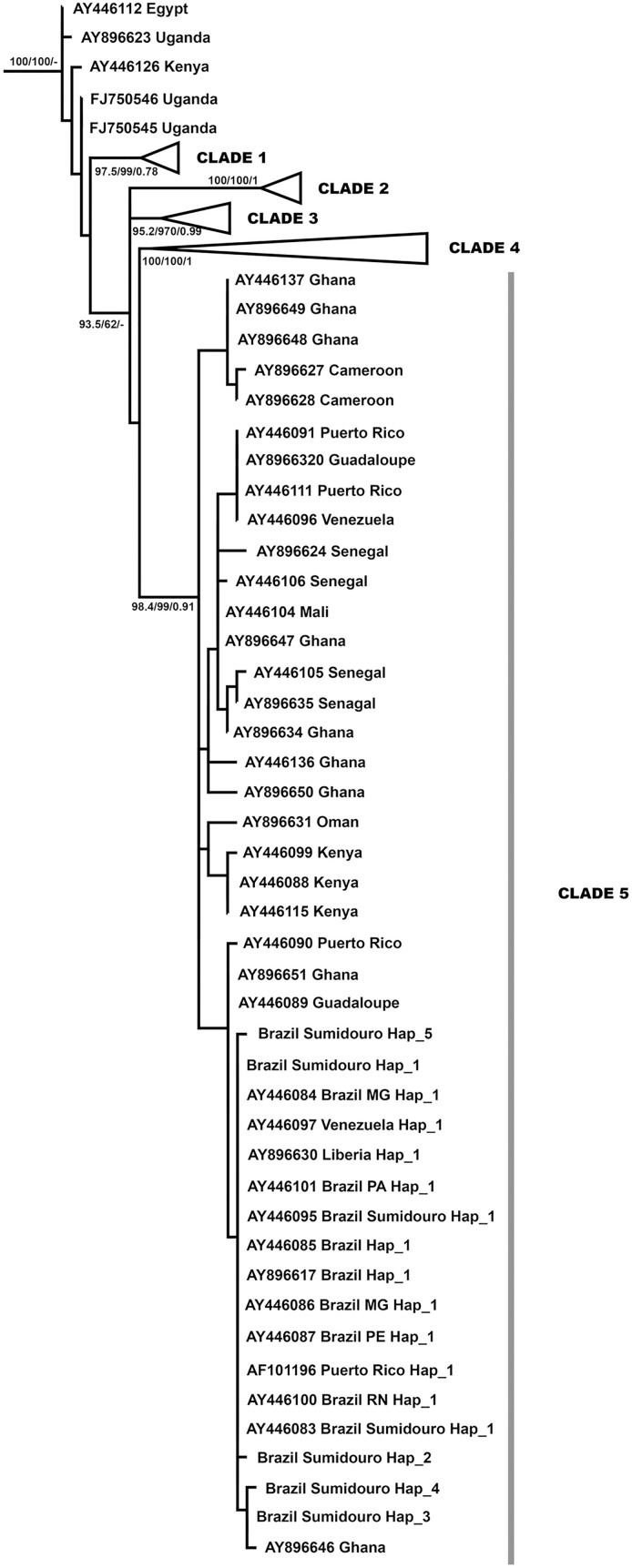
Maximum likelihood phylogenetic tree of *Schistosoma mansoni* partial MT-CO1 sequences from this study and from GenBank, without outgroup sequences. The node values are UFBoot, SH-aLRT and BPP supports.

The tree topologies were similar, with some variations in branch support (Fig 2; S1 Fig). Five *S. mansoni* clades were found. Clade 1 (UFBoot = 97.5; SH-Alrt = 99; BPP = 0.78) included sequences from Madagascar and Zambia. Clade 2 (UFBoot = 100; SH-Alrt = 100; BPP = 1) included sequences from Cameroon, Egypt and Nigeria. Clade 3 (UFBoot =95.2; SH-Alrt = 97; BPP = 0.99) included sequences from Kenya, Mali, Tanzania, and Uganda. Clade 4 (UFBoot = 100; SH-Alrt = 100; BPP = 1) included sequences from Kenya, Madagascar, Tanzania, and Zambia. Clade 5 (UFBoot = 98.4; SH-Alrt = 99; BPP = 0.91) grouped sequences from Cameroon, Ghana, Liberia, Senegal, Mali, Puerto Rico, Venezuela, Guadaloupe, Brazil and Oman.

Among the 122 *S. mansoni* sequences studied, 78 haplotypes were obtained. Among them, only haplotype Hap_1, found in the municipality of Sumidouro, was shared with other localities in Brazil, such as the states of Minas Gerais, Pernambuco, Ceará and Pará; in the Americas, such as Venezuela and Puerto Rico; and in West Africa, such as Liberia. The other haplotypes (Hap_2 to Hap_5) were exclusive to Sumidouro, indicating their local diversity. Haplotype 5 was represented by an *S. mansoni* specimen from *N. squamipes* collected in the Santa Cecília locality, so this haplotype was not included in the population genetic analysis of Sumidouro.

#### Genetic diversity of Sumidouro.

**Fig 3:**
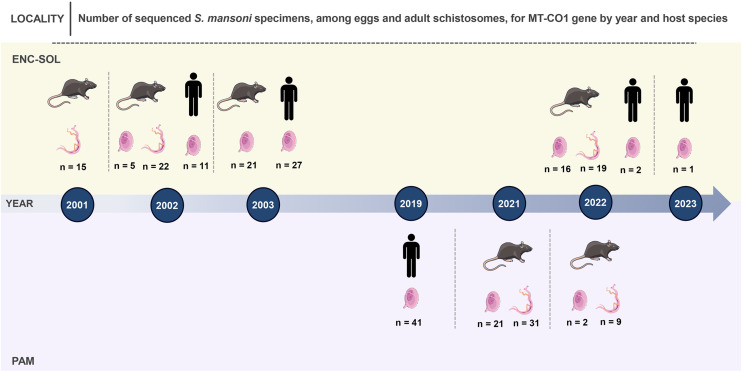
Timeline containing the number of MT-CO1 sequences of *Schistosoma mansoni* eggs and adult schistosomes collected from *N. squamipes* and *S. mansoni* eggs collected from human feces across the time in each locality of Sumidouro, state of Rio de Janeiro state, Brazil. Illustration from NIAID NIH BIOART Source (https://bioart.niaid.nih.gov/bioart/54) and Wikimedia Commons Source (https://commons.wikimedia.org/wiki/File:Schistosoma_mansoni_female.png; https://commons.wikimedia.org/wiki/File:Schistosoma_mansoni_egg_(01).png; https://commons.wikimedia.org/wiki/File:Man_(13779)_-_The_Noun_Project.svg).

We obtained MT-CO1 gene consensus sequences from 243 *S. mansoni* specimens, including eggs and adult schistosomes (S2 Table; Fig 3). We built two different datasets: the first dataset included all sequences of different localities, resulting in 243 taxa and 555 bp, and the second dataset for temporal analysis included only sequences of ENC-SOL, resulting in 139 taxa and 555 bp.

#### Haplotype network of the PAM and ENC-SOL localities.

**Fig 4:**
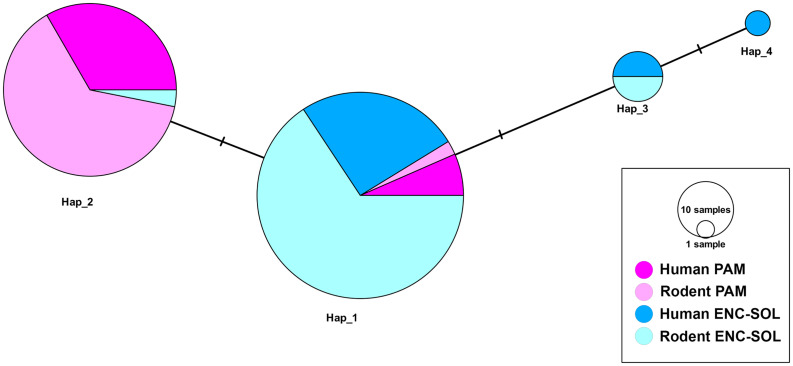
Median-joining network for partial MT-CO1 sequence haplotypes of 243 *Schistosoma mansoni* sequences from the Encanto and Soledade (ENC-SOL) and Pamparrão (PAM) localities in Sumidouro, state of Rio de Janeiro, Brazil. Circle sizes are proportional to haplotype frequencies, and colors represent the definitive hosts of each geographical locality in which haplotypes occur. Each hatch mark along the branches represents one mutation at a step separating haplotypes.

Among 243 partial MT-CO1 gene sequences (555 bp) of *S. mansoni*, we identified four haplotypes with three polymorphic sites, separated by genetic distances of one mutational step. The results of the neutrality deviation tests were not significant, indicating that, this genetic marker, populations are not undergoing population expansion or retraction (Table 2). We observed haplotypic sharing between humans and rodents. Haplotype 1 (Hap_1) was found in all the studied localities and was predominant in ENC-SOL. Haplotype 2 (Hap_2) was found in the localities of PAM and ENC-SOL. In the latter locality, this haplotype was found in only three different sources (two eggs and one adult schistosome) recovered from three *N. squamipes* individuals. Haplotypes 3 and 4 (Hap_3 and Hap_4) were found only in ENC-SOL. Hap_3 was found both in humans and rodents, and Hap_4 was found only in humans (Fig 4).

**Table 2:** Genetic diversity indices and neutrality deviation tests (Tajima’s D and Fu’s Fs) for *Schistosoma mansoni* MT-CO1 sequences from definitive hosts from the PAM and ENC-SOL localities in Sumidouro, state of Rio de Janeiro, Brazil.

	N	H	S	Hd	π	Tajima’s D	Fu’s Fs
Human PAM	41	2	1	0.35122	0.00063	0.71273	1.13918
Rodent PAM	63	2	1	0.09217	0.00017	-0.71756	-0.62788
Human ENC-SOL	41	3	2	0.26585	0.00063	-0.46644	-0.43402
Rodent ENC-SOL	98	3	2	0.15485	0.00029	-0.91369	-1.50607

N: sample size; H: number of haplotypes; S: number of polymorphic sites; Hd: haplotype diversity; π: nucleotide diversity; neutrality deviation tests were not significant (*p* > 0.05)

#### Population structure between localities.

Considering the fixation indices for different localities, the *F*_ST_ values revealed significant genetic differences between localities when each host type was considered separately. Between rodents and humans within and between localities, significant *F*_ST_ values were observed in all comparisons, except between humans and rodents in ENC-SOL (Table 3).

**Table 3:** Pairwise *F*_ST_ values for MT-CO1 sequences of *Schistosoma mansoni* from different definitive hosts (human and rodent) from the PAM and ENC-SOL localities in Sumidouro, state of Rio de Janeiro, Brazil.

	Human ENC-SOL	Rodent ENC-SOL	Human PAM	Rodent PAM
Human ENC-SOL	0.000			
Rodent ENC-SOL	0.013	0.000		
Human PAM	0.620*	0.708*	0.000	
Rodent PAM	0.830*	0.859*	0.117*	0.000

* Significant results (*p *< 0.01).

The AMOVA revealed the greatest variation between localities, which represented 76.41% of the total variation; however, it was not significant (*F*_CT_ = 0.7636, **p* *= 0.345). The genetic variation between humans and rodents within localities was only 1.52% (*F*_SC_ = 0.06495, **p* *= 0.006). The genetic variation between humans and rodents from different localities was 21.85% (*F*_ST_ = 0.78146, *p* = < 0.01). Considering the comparisons between the dataset of eggs and *S. mansoni* adult schistosomes recovered from rodents, the *F*_ST_ values and the AMOVA results revealed no significant genetic differences at any locality.

#### Haplotype network and population structure of the ENC-SOL locality: temporal analysis.

**Fig 5:**
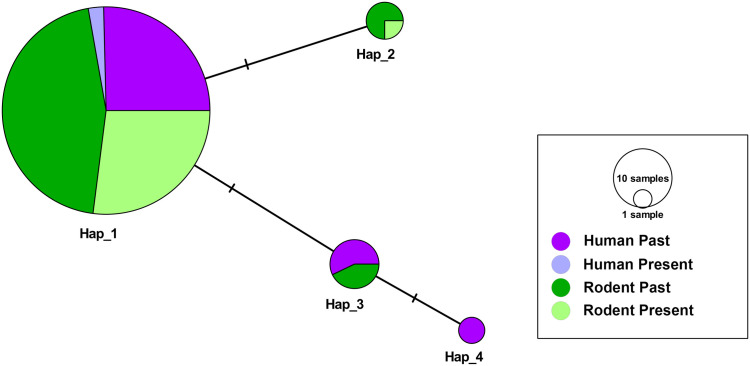
Median-joining network for partial MT-CO1 sequence haplotypes of 139 *S. mansoni* specimens collected between 2001 and 2003 and between 2022 and 2023 in the ENC-SOL locality in Sumidouro, state of Rio de Janeiro, Brazil. Circle sizes are proportional to haplotype frequencies; colors represent the definitive hosts. Each hatch mark along the branch haplotypes represents one mutation at a step separating the haplotypes.

When performing a temporal analysis with only *S. mansoni* specimens from the ENC-SOL locality, we found the same haplotypes recovered for the two localities together (PAM and ENC-SOL) (Fig 5). There was no difference in haplotype frequency across collections (22 years apart). Considering the fixation index for the ENC-SOL dataset, the *F*_ST_ values did not reveal genetic differences between *S. mansoni* specimens at a 22-year interval in the ENC-SOL locality. The AMOVA results revealed greater variation within each sampling period, which represented 97.47% of the total variation (*F*_ST_ = 0.03534, *p* = 0.07).

#### Discriminant analysis of principal components.

**Fig 6:**
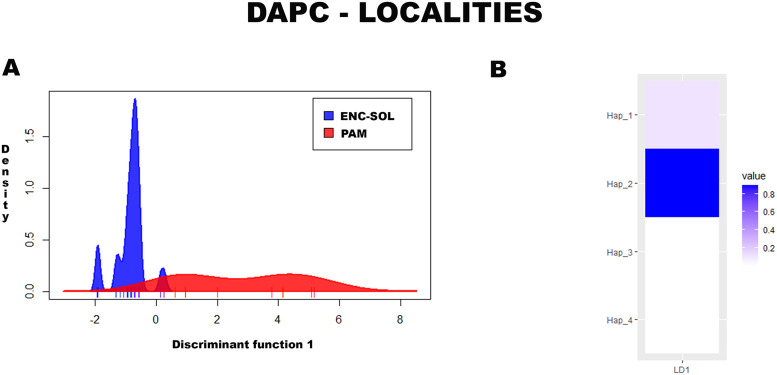
(A) Population clusters for MT-CO1 haplotypes, based on DAPC, showing differences in haplotype frequencies between localities. (B) Haplotype contributions to differences between localities.

In the DAPC for localities, two PCs were retained, explaining approximately 99% of the data. All the haplotypes were correctly classified into their original group. Variation in haplotype frequency between localities was observed (Wilks’ Lambda = 0.19, df = 4, *p* < 0.01), with low overlap between the PAM and ENC-SOL localities (Fig 6A). The haplotype that best discriminated the localities was Hap_2 (Fig 6B).

**Fig 7:**
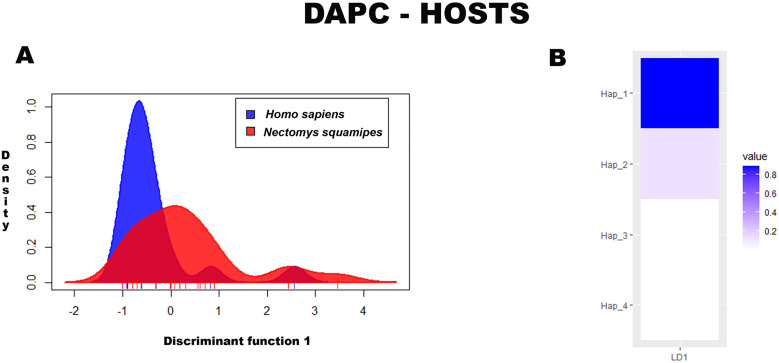
(A) Population clusters for MT-CO1 haplotypes, based on DAPC, showing differences in haplotype frequencies between definitive hosts. (B) Haplotype contributions to differences between definitive hosts.

Regarding hosts, similar to the DAPC for localities, two PCs were retained in the analysis, explaining approximately 99% of the variation in the data. The proportion of haplotypes classified correctly to their original group was approximately 50%. No variation in haplotype frequency between hosts was observed (Wilks’ Lambda = 0.86, df = 4, *p* = 0.22), with a marked overlap observed between humans and rodents (Fig 7A). The haplotype that best discriminated the hosts was Hap_1 (Fig 7B).

**Fig 8:**
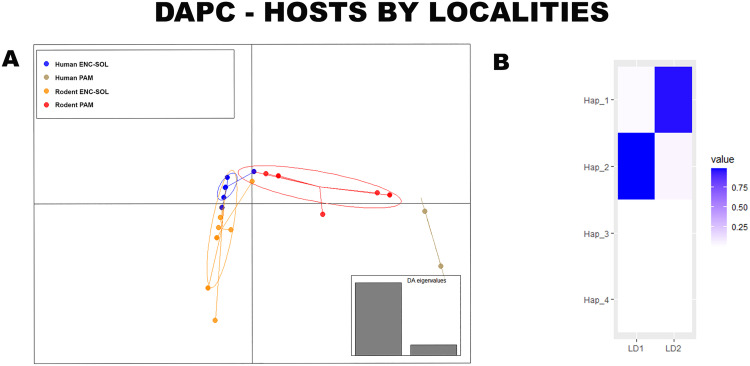
(A) Population clusters for MT-CO1 haplotypes, based on DAPC, showing differences in haplotype frequencies between definitive hosts of different localities. (B) Haplotype contributions to differences between definitive hosts of different localities.

When considering both hosts and localities, two PCs were retained in the DAPC, similar to the previous analyses, representing approximately 99% of the data variation. The proportion of haplotypes classified correctly to their original group was approximately 57%. Variation in the haplotype frequency between hosts in each locality was observed (Wilks’ Lambda = 0.14, df = 12, p < 0.01), with a greater difference in the haplotype frequency of humans in the PAM than in that of rodents in the same locality and in ENC-SOL (Fig 8A). However, it should be taken into account that the sample number of infrapopulations from humans in the PAM was lower than the number of infrapopulations from rodents (S2 Table). There was also low overlap in the frequency of schistosome haplotypes from rodents between PAM and ENC-SOL, with only one rodent from ENC-SOL having a haplotype frequency highly similar to that of schistosomes collected from rodents in the PAM. Schistosomes collected from humans in ENC-SOL presented high similarity in haplotype frequency with those collected from rodents in this locality but low similarity in haplotype frequency with schistosomes collected from rodents in the PAM. Haplotypes 1 and 2 were considered the haplotypes that best discriminated the studied groups (Fig 8B).

### Microsatellite analyses

#### Genetic diversity.

**Fig 9:**
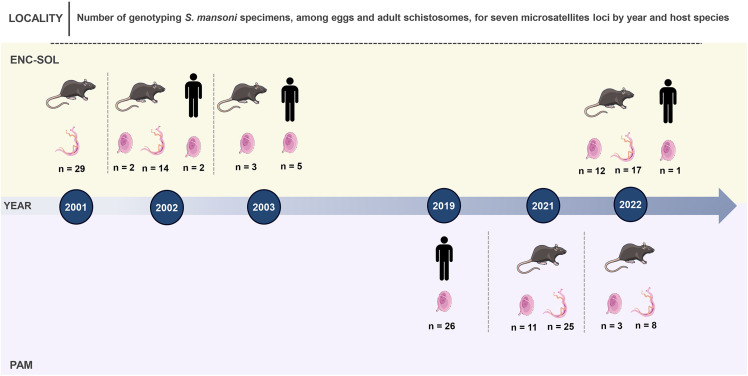
Timeline containing the number of genotyping *Schistosoma mansoni* specimens for seven microsatellite loci among eggs and adult schistosomes collected from *Nectomys squamipes* and *Schistosoma mansoni* eggs collected from human feces over time at each locality in Sumidouro, Rio de Janeiro state, Brazil. Illustration from NIAID NIH BIOART Source (https://bioart.niaid.nih.gov/bioart/54) and Wikimedia Commons Source (https://commons.wikimedia.org/wiki/File:Schistosoma_mansoni_female.png; https://commons.wikimedia.org/wiki/File:Schistosoma_mansoni_egg_(01).png; https://commons.wikimedia.org/wiki/File:Man_(13779)_-_The_Noun_Project.svg).

Seven microsatellite loci were used for genotyping 158 *S. mansoni* specimens, among adult schistosomes and egg samples (S3 Table; Fig 9). Null alleles were detected for locus 15J15 only in the ENC-SOL rodent group, for locus SM13–478 in all groups and for locus SMMS16 in the PAM human and ENC-SOL rodent groups. We detected significant linkage disequilibrium between some pairs of loci (S4 Table) and observed deviation from HWE in some loci in all studied groups (S5 Table). The descriptive statistics of the seven microsatellite loci of *Schistosoma mansoni* are presented in Table 4. All the loci were polymorphic, and the number of alleles ranged from 3 to 13 (Table 4). The *F*_*IS*_ values were positive in all the groups studied, except in the Human PAM group (Table 4).

**Table 4:** Descriptive statistics of genetic variation of seven microsatellite loci of *Schistosoma mansoni* of different definitive hosts from the PAM and ENC-SOL localities in Sumidouro, state of Rio de Janeiro, Brazil.

		1F8A	15J15A	29E6A	SM13–478	SMMS 3	SMMS 16	SMMS 18	*F*_*IS*_ (*p *= 0.0018)
Rodent PAM	***N***	46	46	45	46	47	47	47	0.111 (*p = *0.0018)
	***Na***	6	9	5	6	6	5	3	
	***H***__***O***__	0.739	0.630	0.467	0.522	0.489	0.574	0.532	
	***H***_***E***_	0.698	0.739	0.576	0.682	0.568	0.679	0.449	
Human PAM	***N***	23	25	24	25	26	26	26	0.051 (*p = *0.1536)
	***Na***	7	6	5	5	5	4	5	
	***H***_***O***_	0.783	0.480	1.000	0.440	0.500	0.385	0.731	
	***H***_***E***_	0.689	0.571	0.610	0.751	0.615	0.663	0.556	
Rodent ENC-SOL	***N***	76	77	77	77	77	77	75	0.121 (*p = *0.0018)
	***Na***	8	9	8	13	12	9	9	
	***H***_***O***_	0.776	0.701	0.468	0.675	0.753	0.494	0.747	
	***H***_***E***_	0.761	0.825	0.535	0.838	0.792	0.702	0.755	
Human ENC-SOL	***N***	8	7	8	8	8	7	8	0.336 (*p = *0.0018)
	***Na***	6	4	3	7	3	5	3	
	***H***_***O***_	0.625	0.429	0.375	0.500	0.250	0.429	0.500	
	***H***_***E***_	0.719	0.704	0.320	0.820	0.406	0.745	0.555	

*N*: number of individuals per group; *Na:* number of alleles per locus; *H*_*O*_: observed heterozygosity; *H*_*E*_: expected heterozygosity; *F*_*IS*_: inbreeding coefficient (*p-*value - Bonferroni correction).

#### Population structure between localities.

Considering the fixation index for the different localities, the microsatellite *F*_ST_ values revealed significant genetic differences in all pairwise comparisons (**p* *< 0.01) (Table 5). The AMOVA results revealed that the greatest variation observed was between humans and rodents from different localities, which represented 87.89% of the total variation (*F*_ST_ = 0.121, **p* *< 0.01). The genetic variation between localities was 9.44% (*F*_CT_ = 0.094; **p* *< 0.01). The genetic variation between humans and rodents within localities was 2.66% (*F*_SC_ = 0.03; **p* *< 0.01). Considering the fixation index between localities in the 2019–2023 period, gathering the data of the two definitive hosts, the *F*_ST_ value revealed significant genetic differences between localities (*F*_ST_ = 0.15885; *p* = < 0.01).

**Table 5:** Pairwise *F*_ST_ values for microsatellite loci of *Schistosoma mansoni* from different definitive hosts from the PAM and ENC-SOL localities in Sumidouro, state of Rio de Janeiro, Brazil.

	Rodent PAM	Human PAM	Rodent ENC-SOL	Human ENC-SOL
Rodent PAM	0.000			
Human PAM	0.019*	0.000		
Rodent ENC-SOL	0.118*	0.114*	0.000	
Human ENC-SOL	0.123*	0.127*	0.047*	0.000

* Significant results (*p *< 0.01).

**Fig 10:**
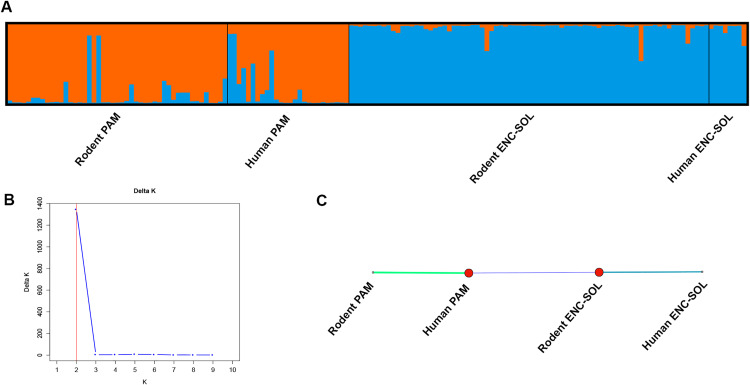
(A) Population structure based on microsatellite genotyping of 158 *Schistosoma mansoni* individuals for K = 2 organized by *Nectomys squamipes* and human hosts of the Pamparrão locality and *Nectomys squamipes* and human hosts of the Encanto locality, municipality of Sumidouro, state of Rio de Janeiro. (B) ΔK value for K = 2. (C) Automatically thresholded network (0.13) of *Schistosoma mansoni* populations, generated with EDENetwork software, based on *F*_ST_. The edge weight is inversely proportional to the *F*_ST_ value; thicker edges denote lower pairwise *F*_ST_; edges color tracks thickness.

EDENetwork analysis revealed thicker edges in the network between the Rodent PAM and Human PAM groups and between the Rodent ENC-SOL and Human ENC-SOL groups, denoting a connectedness between these groups (Fig 10C). Conversely, the network revealed narrow edges between the PAM and ENC-SOL groups, suggesting restricted gene flow between these two localities (Fig 10C).

Bayesian analysis of population structure clustered individuals from the four studied groups of *S. mansoni* into two clusters. The ΔK method revealed two clusters (Fig 10B) as the most likely population structure, with individuals clustered according to these localities (PAM and ENC-SOL) (Fig 10A). Structure cluster graphs of the studied groups of *S. mansoni* in Sumidouro, with minor and major modes for K = 3–10, are presented in the (S2 Fig.).

#### Population structure of the ENC-SOL locality: temporal analysis.

Considering the fixation index for the ENC-SOL dataset, the *F*_ST_ value revealed a very low genetic difference between *S. mansoni* specimens over a 22-year interval in the ENC-SOL locality (*F*_ST_ = 0.04045, p = < 0.01) indicating no genetic structure over time.

## Discussion

### Phylogenetic relationships between Sumidouro and other endemic areas

Phylogenetic analyses of MT-CO1 suggest that the evolutionary origin of *S. mansoni* lies in East Africa, where the greatest genetic diversity is observed [40,80]. The phylogenetic reconstructions that include our *S. mansoni* sequences from Sumidouro and sequences deposited in Genbank corroborate these hypotheses, identifying five lineages associated with the geographic distribution of samples without associations with specific host species. The data support the hypotheses that *S. mansoni* was introduced into South America via the transatlantic slave trade between the 15th and 19th centuries, with haplotypes from Sumidouro closely related to those from West Africa [40,80]. Hap_1, found in Sumidouro, was shared with other South American localities and a single West African (Liberia) locality. The absence of Hap_1 in other African localities may reflect genetic differentiation due to low gene flow between African and South American *S. mansoni* populations or local differentiation of other haplotypes in Africa. However, most of the sequences obtained from these locations were from strains kept in the laboratory, which have low genetic diversity and may justify haplotype sharing [40]. Additionally, four haplotypes unique to Sumidouro were identified, with three being less frequent. Studies suggest that rare haplotypes may result from local differentiation [80,81].

Although new haplotypes have been identified in Sumidouro, genetic diversity is still low in Brazil compared with the genetic diversity rates observed in African countries [40,80]. This low diversity has been attributed to the founder effect resulting from the recent introduction of *S. mansoni* into South America [40].

### Population structure

This study investigated the population structure of *S. mansoni* in the municipality of Sumidouro, focusing on two endemic localities. The aim of this study was to assess genetic differentiation between the definitive hosts (humans and water-rats) using the MT-CO1 gene and seven microsatellite loci. The genetic structure analyses, which were based on both the MT-CO1 gene and microsatellite loci used, were consistent and indicated an absence of isolation between *S. mansoni* populations from humans and water rats. This was evidenced by haplotypic sharing between isolates from the two host species, suggesting multi-host transmission in this region. These findings support the role of *N. squamipes* in maintaining the parasite life cycle, which contributes to human infections.

The results indicated significant gene flow between *S. mansoni* populations in different hosts, with no isolation observed between chronotypes. Among the 243 individuals sequenced for the MT-CO1 gene, four haplotypes were identified in the PAM and ENC-SOL localities, with haplotypes 1, 2, and 3 being shared between humans and water-rats and haplotype 4 being found only in humans. Haplotypes 3 and 4 were exclusive to the ENC-SOL locality, whereas haplotypes 1 and 2 occurred in both localities. AMOVA of the MT-CO1 gene and microsatellite loci revealed low genetic variation between definitive hosts within each locality. The consumption of the intermediate host, the snail *Biomphalaria glabrata*, by rodents, as observed in laboratory experiments, may contribute to parasite homogenization across chronotypes in the definitive host.

However, when comparing hosts from different localities, genetic differentiation was detected through DAPC and *F*_ST_ analyses, with significant values indicating a geographical structure in the *S. mansoni* populations. Bayesian population structure analysis confirmed the existence of two distinct genetic groups based on locality, with minimal admixture between them.

In ENC-SOL, the *F*_ST_ values were low for both genetic markers between humans and rodents. Similarly, the PAM showed significant, although low, *F*_ST_ values, suggesting gene flow between human and rodent *S. mansoni* populations. DAPC analysis further supported these findings, showing no variation in haplotypes between humans and rodents within localities. Temporal analysis over a 22-year interval in ENC-SOL did not indicate population differentiation, suggesting that the observed geographic structure between ENC-SOL and PAM was not due to temporal factors.

A study by Catalano et al. [7] in Senegal, West Africa, demonstrated the zoonotic nature of *S. mansoni* by showing haplotype sharing between humans and wild rodents, similar to the present findings in Sumidouro. Both studies support the role of wild rodents as reservoirs, contributing to transmission in these different regions. Similarly, Long et al. [82] identified genetic structure among *S. mansoni* populations in human communities in Bahia, Brazil, with limited gene flow between isolated populations. These findings align with our study, where geographic proximity did not prevent low gene flow, likely because of human movement between localities rather than rodent dispersal, which is confined to riverbanks [11,83]. In contrast, Standley et al. [81] did not observe geographic genetic structure among *S. mansoni* isolates from humans across Lake Victoria in Kenya, Tanzania, and Uganda, attributing the lack of differentiation to human migration, which homogenized *S. mansoni* populations along the lake’s shorelines.

No genetic differences were detected between the egg and adult stages of *S. mansoni*, confirming that eggs accurately represent the gene pool of the parasite infrapopulations. This is consistent with findings by Shrivastava et al. [84], who reported no genetic differentiation between larval and adult stages in *S. japonicum* from wild rodents.

Although microsatellite markers used in this study and others discussed here allow inferences about fine-scale kinship patterns among schistosomes, whole-genomic approaches have been used to improve the resolution of evolutionary aspects and population structure of schistosomes [85–87]. These population genomic studies involving schistosomes have provided a more detailed resolution of the population structure of these parasites, clarifying the transmission patterns and the response to the treatment with praziquantel because it is possible to estimate the degree of kinship between larval stages of the parasite between hosts and among transmission sites. This genomic approach highlighted hotspots of persistent transmission and reemergence of the disease in endemic areas where mass drug administration programs are adopted [86–88]. In addition, the presence of wild reservoir hosts highlights attention for studying the genetic variation in *S. mansoni* populations that may be associated with different chronotypes from humans and wild reservoir [88].

In conclusion, this study confirmed that wild rodents, particularly *N. squamipes*, contribute to the maintenance of the *S. mansoni* cycle in Sumidouro, acting as a wild reservoir of schistosomiasis. Despite possible pre-zygotic isolation, no genetic evidence of host isolation was observed.

These results highlight the importance of considering rodents in disease control programs and support the adoption of the One Health approach. The presence of infected wild rodents in endemic areas should be further investigated, as they can serve as indicators of local transmission hotspots.

## Supporting information

S1 Fig Maximum likelihood phylogenetic tree of *Schistosoma mansoni* partial MT-CO1 sequences from this study from GenBank and the outgroup. The node values are UFBoot, SH-aLRT and BPP supports.
(TIF)

S2 Fig Structure cluster graphs of three *Schistosoma mansoni* groups in Sumidouro with minor and major modes for the data. The runs were performed for K = 1–10 with 10 replicates, and division for runs by mode is provided below the graphs.
(TIF)

S1 Table GenBank accession number, geographical locality, host, and reference sequences of the GenBank MT-CO1 partial sequences of *Schistosoma mansoni* and the outgroup (*Schistosoma rodhaini*) used in this study. (DOCX)

S2 Table Number of MT-CO1 sequences of *Schistosoma mansoni* eggs and adult schistosomes collected from *Nectomys squamipes* and *Schistosoma mansoni* eggs collected from human feces over time at each locality in Sumidouro, Rio de Janeiro state, Brazil. (DOCX)

S3 Table Number of genotyping *Schistosoma mansoni* specimens for seven microsatellite loci among eggs and adult schistosomes collected from *Nectomys squamipes* and *Schistosoma mansoni* eggs collected from human feces over time at each locality in Sumidouro, Rio de Janeiro state, Brazil. (DOCX)

S4 Table Probability of linkage disequilibrium for pairs of loci per population of *Schistosoma mansoni* generated by Arlequin 3.5.2.2. (DOCX)

S5 Table Results of the Hardy–Weinberg equilibrium (HWE) test evaluated with GenAIEx version 6.503 using chi-square test. (DOCX)
