# Supplementary figures and images for: Genetic analysis of Schistosoma mansoni in a low-transmission area in Brazil suggests population sharing between wild-hosts and humans and geographical isolation
Source: PLoS Negl Trop Dis. 2025 Aug 11;19(8):e0013379. doi: 10.1371/journal.pntd.0013379 (PMC12338815; doi:10.1371/journal.pntd.0013379)

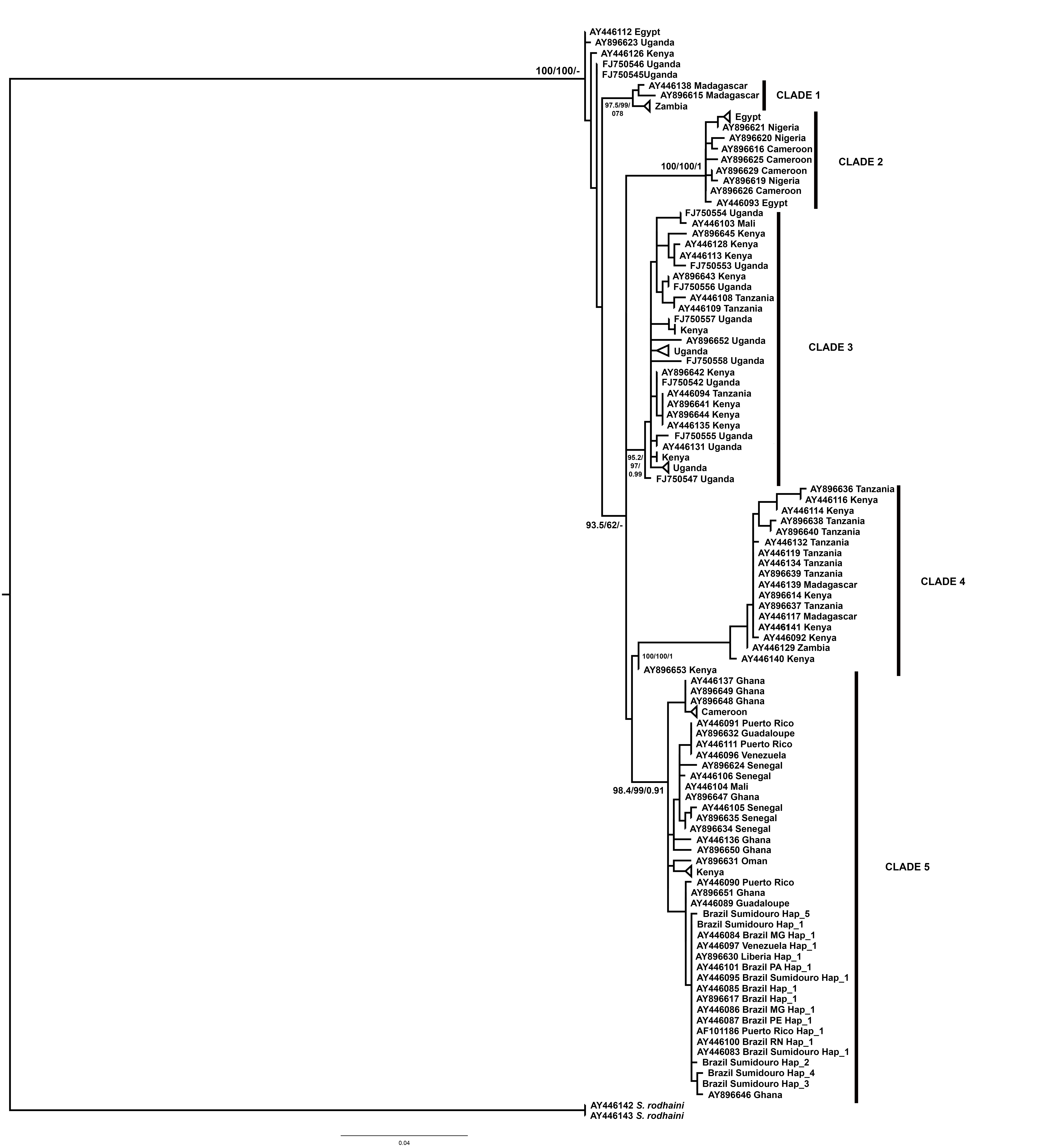

Supplement: S1 Fig — The node values are UFBoot, SH-aLRT and BPP supports. (TIF) [file pntd.0013379.s001.tif]

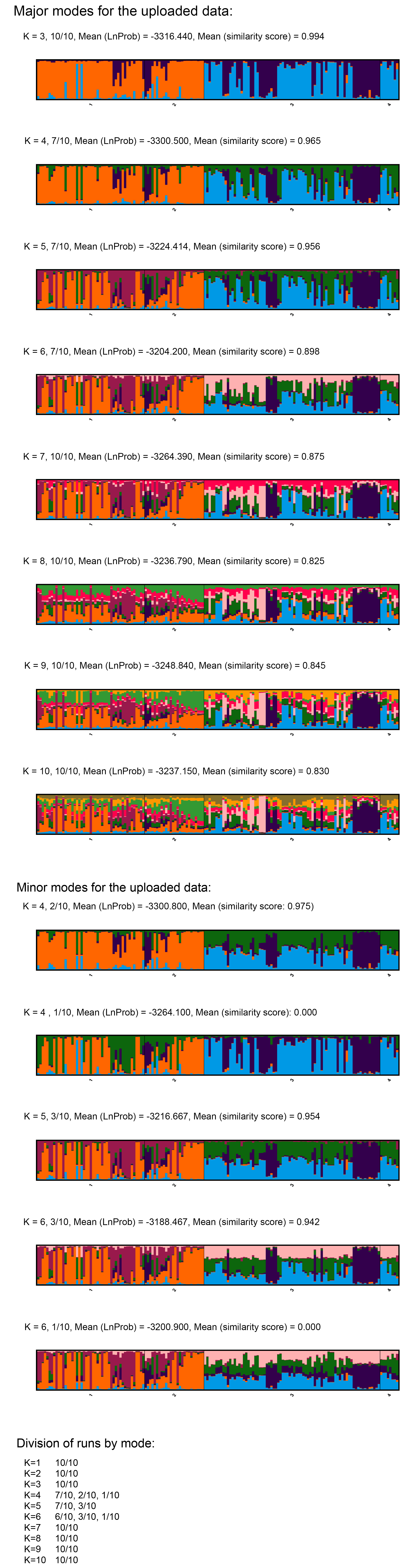

Supplement: S2 Fig — The runs were performed for K = 1–10 with 10 replicates, and division for runs by mode is provided below the graphs. (TIF) [file pntd.0013379.s002.tif]
