# Supplementary material for: Genetic analysis of Schistosoma mansoni in a low-transmission area in Brazil suggests population sharing between wild-hosts and humans and geographical isolation
Source: PLoS Negl Trop Dis. 2025 Aug 11;19(8):e0013379. doi: 10.1371/journal.pntd.0013379 (PMC12338815; doi:10.1371/journal.pntd.0013379)
Supplement: S1 Table — (DOCX) [file pntd.0013379.s003.docx]

**S1 Table.** GenBank accession number, geographical locality, host, and reference sequences of the GenBank MT-CO1 partial sequences of *Schistosoma mansoni* and the outgroup (*Schistosoma rodhaini*) used in this study.

| Accession number | Locality | Host | References |
| --- | --- | --- | --- |
| Hap_1 | Brazil: Sumidouro | *Homo sapiens; Nectomys squamipes* | Present study |
| Hap_2 | Brazil: Sumidouro | *Homo sapiens; Nectomys squamipes* | Present study |
| Hap_3 | Brazil: Sumidouro | *Homo sapiens; Nectomys squamipes* | Present study |
| Hap_4 | Brazil: Sumidouro | *Homo sapiens* | Present study |
| Hap_5 | Brazil: Santa Cecília, Sumidouro | *Nectomys squamipes* | Present study |
| AY446122 | Egypt: Mansafire, Menia Governorate | *Homo sapiens* | Morgan et al. [40] |
| AY446123 | Egypt: Mansafire, Menia Governorate | *Biomphalaria alexandrina* | Morgan et al. [40] |
| AY446124 | Egypt: Mansafire, Menia Governorate | *Biomphalaria alexandrina* | Morgan et al. [40] |
| AY896620 | Nigeria |  | Morgan et al. [40] |
| AY896616 | Cameroon |  | Morgan et al. [40] |
| AY896629 | Cameroon |  | Morgan et al. [40] |
| AY896619 | Nigeria |  | Morgan et al. [40] |
| AY446093 | Egypt: long term lab isolate SSCP supplies |  | Morgan et al. [40] |
| AY896621 | Nigeria |  | Morgan et al. [40] |
| AY446120 | Egypt: Alzahraa, Kafr El Sheikh Governorate | *Homo sapiens* | Morgan et al. [40] |
| AY446121 | Egypt: Alzahraa, Kafr El Sheikh Governorate | *Homo sapiens* | Morgan et al. [40] |
| AY446125 | Egypt: Mansafire, Menia Governorate | *Biomphalaria alexandrina* | Morgan et al. [40] |
| AY896618 | Egypt |  | Morgan et al. [40] |
| AY896626 | Cameroon |  | Morgan et al. [40] |
| AY896625 | Cameroon |  | Morgan et al. [40] |
| FJ750554 | Uganda | *Homo sapiens* | Stothard et al. [41] |
| AY446103 | Kenya: Kisumu | *Biomphalaria sudanica* | Morgan et al. [40] |
| AY896636 | Tanzania |  | Morgan et al. [40] |
| AY446116 | Kenya: Near Machakos, Mwanyani River | *Biomphalaria pfeifferi* | Morgan et al. [40] |
| AY446114 | Kenya: Near Machakos, Mwanyani River | *Biomphalaria pfeifferi* | Morgan et al. [40] |
| AY446092 | Kenya: Near Machakos, Mwanyani River | *Biomphalaria pfeifferi* | Morgan et al. [40] |
| AY446132 | Tanzania: Kimamba, Darangani, Ilula Town | *Biomphalaria pfeifferi* | Morgan et al. [40] |
| AY446119 | Tanzania: Kimamba, Darangani, Ilula Town | *Biomphalaria pfeifferi* | Morgan et al. [40] |
| AY446141 | Kenya: Kalina Koi, Kibwezi | *Papio cyanocephalus* | Morgan et al. [40] |
| AY896637 | Tanzania |  | Morgan et al. [40] |
| AY446117 | Madagascar: Antsirabe | *Biomphalaria pfeifferi* | Morgan et al. [40] |
| AY446139 | Madagascar: Mananjary Town | *Homo sapiens* | Morgan et al. [40] |
| AY896614 | Kenya |  | Morgan et al. [40] |
| AY896639 | Tanzania |  | Morgan et al. [40] |
| AY446129 | Zambia: Kariba Dam | *Homo sapiens* | Morgan et al. [40] |
| AY446134 | Tanzania: Tunduma, Safisha stream | *Biomphalaria pfeifferi* | Morgan et al. [40] |
| AY446140 | Kenya: Kalina Koi, Kibwezi | *Papio cyanocephalus* | Morgan et al. [40] |
| AY896638 | Tanzania |  | Morgan et al. [40] |
| AY896640 | Tanzania |  | Morgan et al. [40] |
| FJ750558 | Uganda | *Homo sapiens* | Stothard et al. [41] |
| FJ750555 | Uganda | *Homo sapiens* | Stothard et al. [41] |
| FJ750547 | Uganda | *Homo sapiens* | Stothard et al. [41] |
| FJ750549 | Uganda | *Homo sapiens* | Stothard et al. [41] |
| AY896652 | Uganda |  | Morgan et al. [40] |
| FJ750550 | Uganda | *Homo sapiens* | Stothard et al. [41] |
| FJ750551 | Uganda | *Homo sapiens* | Stothard et al. [41] |
| FJ750557 | Uganda | *Homo sapiens* | Stothard et al. [41] |
| AY446102 | Kenya: Kisumu | *Biomphalaria sudanica* | Morgan et al. [40] |
| AY446107 | Kenya: Kisumu | *Biomphalaria sudanica* | Morgan et al. [40] |
| AY446127 | Kenya: Mtito River | *Biomphalaria pfeifferi* | Morgan et al. [40] |
| AY446133 | Kenya: Mata village, Taveta | *Homo sapiens* | Morgan et al. [40] |
| FJ750552 | Uganda | *Homo sapiens* | Stothard et al. [41] |
| AY446098 | Uganda: Lake Albert | *Biomphalaria sudanica* | Morgan et al. [40] |
| AY896642 | Kenya |  | Morgan et al. [40] |
| FJ750542 | Uganda | *Homo sapiens* | Stothard et al. [41] |
| AY446094 | Tanzania: Mwanza, Mirongo stream | *Biomphalaria sudanica* | Morgan et al. [40] |
| AY446135 | Kenya: Homa Bay | *Biomphalaria sudanica* | Morgan et al. [40] |
| AY896641 | Kenya |  | Morgan et al. [40] |
| AY896644 | Kenya |  | Morgan et al. [40] |
| AY446131 | Uganda: Lake Albert | *Biomphalaria sudanica* | Morgan et al. [40] |
| FJ750543 | Uganda | *Homo sapiens* | Stothard et al. [41] |
| AY446118 | Uganda: Lake Albert | *Biomphalaria sudanica* | Morgan et al. [40] |
| AY896622 | Uganda |  | Morgan et al. [40] |
| FJ750548 | Uganda | *Homo sapiens* | Stothard et al. [41] |
| FJ750544 | Uganda | *Homo sapiens* | Stothard et al. [41] |
| AY896645 | Kenya |  | Morgan et al. [40] |
| AY446128 | Kenya: Mathoungouta, Mwea | *Biomphalaria pfeifferi* | Morgan et al. [40] |
| AY446113 | Kenya: Near Machakos, Mwanyani River | *Biomphalaria pfeifferi* | Morgan et al. [40] |
| FJ750553 | Uganda | *Homo sapiens* | Stothard et al. [41] |
| AY896643 | Kenya |  | Morgan et al. [40] |
| FJ750556 | Uganda | *Homo sapiens* | Stothard et al. [41] |
| AY446108 | Tanzania: Kaseni-Shuleni, Ukerewe Is, Lake Victoria | *Biomphalaria choanomphala* | Morgan et al. [40] |
| AY446109 | Tanzania: Nansio, Ukerewe Is, Lake Victoria | *Biomphalaria sudanica* | Morgan et al. [40] |
| AY446138 | Madagascar: Ihosy | *Biomphalaria pfeifferi* | Morgan et al. [40] |
| AY896615 | Madagascar |  | Morgan et al. [40] |
| AY896633 | Zambia |  | Morgan et al. [40] |
| AY446110 | Zambia: Kariba Dam | *Homo sapiens* | Morgan et al. [40] |
| AY446130 | Zambia: Kariba Dam | *Homo sapiens* | Morgan et al. [40] |
| AY446112 | Kenya: Near Machakos, Mwanyani River | *Biomphalaria pfeifferi* | Morgan et al. [40] |
| AY896623 | Uganda |  | Morgan et al. [40] |
| AY446126 | Kenya: Machakos Town, Musilili stream | *Biomphalaria pfeifferi* | Morgan et al. [40] |
| FJ750546 | Uganda | *Homo sapiens* | Stothard et al. [41] |
| FJ750545 | Uganda | *Homo sapiens* | Stothard et al. [41] |
| AY896653 | Kenya |  | Morgan et al. [40] |
| AY896631 | Oman |  | Morgan et al. [40] |
| AY446137 | Ghana: Accra | *Biomphalaria pfeifferi* | Morgan et al. [40] |
| AY896648 | Ghana |  | Morgan et al. [40] |
| AY896649 | Ghana |  | Morgan et al. [40] |
| AY896627 | Cameroon |  | Morgan et al. [40] |
| AY896628 | Cameroon |  | Morgan et al. [40] |
| AY446091 | Guadeloupe: DFO, Grand-Terre | *Rattus rattus* | Morgan et al. [40] |
| AY446096 | Venezuela: long term lab isolate Italo M. Cesari |  | Morgan et al. [40] |
| AY446111 | Puerto Rico: PR1 long term lab isolate Eric S. Loker |  | Morgan et al. [40] |
| AY896632 | Guadeloupe |  | Morgan et al. [40] |
| AY446105 | Senegal: Richard Toll, Ndiangue | *Biomphalaria pfeifferi* | Morgan et al. [40] |
| AY446106 | Senegal: Richard Toll, Ndombo | *Biomphalaria pfeifferi* | Morgan et al. [40] |
| AY446104 | Mali: Bamako, Farako River | *Biomphalaria pfeifferi* | Morgan et al. [40] |
| AY896647 | Ghana |  | Morgan et al. [40] |
| AY896634 | Senegal |  | Morgan et al. [40] |
| AY896635 | Senegal |  | Morgan et al. [40] |
| AY896624 | Senegal |  | Morgan et al. [40] |
| AY446136 | Ghana: Accra | *Biomphalaria pfeifferi* | Morgan et al. [40] |
| AY896650 | Ghana |  | Morgan et al. [40] |
| AY896646 | Ghana |  | Morgan et al. [40] |
| AY446090 | Puerto Rico: Caguas Valley | *Homo sapiens* | Morgan et al. [40] |
| AY446099 | Kenya: Kitui Town | *Biomphalaria pfeifferi* | Morgan et al. [40] |
| AY446083 | Brazil: Sumidouro, Rio de Janeiro | *Homo sapiens* | Morgan et al. [40] |
| AY446084 | Brazil: Dionisio, Minas Gerais | *Biomphalaria glabrata* | Morgan et al. [40] |
| AY446086 | Brazil: Sabara, Minas Gerais | *Biomphalaria glabrata* | Morgan et al. [40] |
| AY446087 | Brazil: Recife, Pernambuco | *Biomphalaria straminea* | Morgan et al. [40] |
| AY446095 | Brazil: Sumidouro, Rio de Janeiro | *Neotomys sp.* | Morgan et al. [40] |
| AY446097 | Venezuela: long term lab isolate R. Nino Incani |  | Morgan et al. [40] |
| AY446100 | Brazil: CMO, Ceara-Mirim, Rio Grande del Norte, long term lab isolate Lygia R. Correa |  | Morgan et al. [40] |
| AY446101 | Brazil: Belem, Para, long term lab isolate Lygia R. Correa |  | Morgan et al. [40] |
| AY896617 | Brazil |  | Morgan et al. [40] |
| AY896630 | Liberia |  | Morgan et al. [40] |
| AF101196 |  |  | Unpublished |
| AY446085 | Brazil: Corrego de Cafe | *Biomphalaria glabrata* | Morgan et al. [40] |
| AY896651 | Ghana |  | Morgan et al. [40] |
| AY446089 | Guadeloupe: Grand Camp, Basse-Terre | *Homo sapiens* | Morgan et al. [40] |
| AY446088 | Kenya: Makueni, Makindu Town | *Biomphalaria pfeifferi* | Morgan et al. [40] |
| AY446115 | Kenya: Near Machakos, Mwanyani River | *Biomphalaria pfeifferi* | Morgan et al. [40] |
| AY446142 (outgroup) | Kenya: Kisumu | *Biomphalaria sudanica* | Morgan et al. [40] |
| AY446143 (outgroup) | Kenya: Homa Bay, Lake Victoria | *Biomphalaria sudanica* | Morgan et al. [40] |
