# Supplementary material for: Genetic analysis of Schistosoma mansoni in a low-transmission area in Brazil suggests population sharing between wild-hosts and humans and geographical isolation
Source: PLoS Negl Trop Dis. 2025 Aug 11;19(8):e0013379. doi: 10.1371/journal.pntd.0013379 (PMC12338815; doi:10.1371/journal.pntd.0013379)
Supplement: S2 Table — (DOCX) [file pntd.0013379.s004.docx]

**S2 Table.** Number of MT-CO1 sequences of *Schistosoma mansoni* eggs and adult schistosomes collected from *Nectomys squamipes* and *Schistosoma mansoni* eggs collected from human feces over time at each locality in Sumidouro, Rio de Janeiro state, Brazil.

| YEAR | LOCALITY | HOST | INFRAPOPULATION | EGGS | SCHISTOSOMES | N |  |
| --- | --- | --- | --- | --- | --- | --- | --- |
| 2001 | ENC-SOL | *N. squamipes* | 4 | - | 15 | 15 |  |
| 2002 | ENC-SOL | *N. squamipes* | 4 | 5 | 22 | 27 |  |
|  | ENC-SOL | Human | 7 | 11 | - | 11 |  |
| 2003 | ENC-SOL | *N. squamipes* | 4 | 21 | - | 21 |  |
|  | ENC-SOL | Human | 9 | 27 | - | 27 |  |
| 2019 | PAM | Human | 2 | 41 | - | 41 |  |
| 2021 | PAM | *N. squamipes* | 8 | 21 | 31 | 52 |  |
| 2022 | PAM | *N. squamipes* | 1 | 2 | 9 | 11 |  |
| 2022 | ENC-SOL | Human | 1 | 2 | - | 2 |  |
|  | ENC-SOL | *N. squamipes* | 2 | 16 | 7 | 23 |  |
| 2022 | ENC-SOL | *N. squamipes* | 1 | - | 12 | 12 |  |
| 2023 | ENC-SOL | Human | 1 | 1 | - | 1 |  |
