# Supplementary material for: Genetic analysis of Schistosoma mansoni in a low-transmission area in Brazil suggests population sharing between wild-hosts and humans and geographical isolation
Source: PLoS Negl Trop Dis. 2025 Aug 11;19(8):e0013379. doi: 10.1371/journal.pntd.0013379 (PMC12338815; doi:10.1371/journal.pntd.0013379)
Supplement: S3 Table — (DOCX) [file pntd.0013379.s005.docx]

**S3 Table.** Number of genotyping *Schistosoma* *mansoni* specimens for seven microsatellite loci among eggs and adult schistosomes collected from *Nectomys* *squamipes* and *Schistosoma* *mansoni* eggs collected from human feces over time at each locality in Sumidouro, Rio de Janeiro state, Brazil.

| **YEAR** | **LOCALITY** | **HOST** | **INFRAPOPULATION** | **EGGS** | **SCHISTOSOMES** | **SSR (N)** |
| --- | --- | --- | --- | --- | --- | --- |
| **2001** | ENC-SOL | *N. squamipes* | 5 | - | 29 | 29 |
| **2002** | ENC-SOL | *N. squamipes* | 3 | 2 | 14 | 16 |
|  | ENC-SOL | Human | 2 | 2 | - | 2 |
| **2003** | ENC-SOL | *N. squamipes* | 2 | 3 | - | 3 |
|  | ENC-SOL | Human | 3 | 5 | - | 5 |
| **2019** | PAM | Human | 2 | 26 | - | 26 |
| **2021** | PAM | *N. squamipes* | 7 | 11 | 25 | 36 |
| **2022** | PAM | *N. squamipes* | 1 | 3 | 8 | 11 |
| **2022** | ENC-SOL | Human | 1 | 1 | - | 1 |
|  | ENC-SOL | *N. squamipes* | 2 | 12 | 7 | 19 |
| **2022** | ENC-SOL | *N. squamipes* | 1 | - | 10 | 10 |
