# Supplementary material for: Genetic analysis of Schistosoma mansoni in a low-transmission area in Brazil suggests population sharing between wild-hosts and humans and geographical isolation
Source: PLoS Negl Trop Dis. 2025 Aug 11;19(8):e0013379. doi: 10.1371/journal.pntd.0013379 (PMC12338815; doi:10.1371/journal.pntd.0013379)
Supplement: S4 Table — (DOCX) [file pntd.0013379.s006.docx]

**S4 Table:** Probability of linkage disequilibrium for pairs of loci per population of *Schistosoma mansoni* generated by Arlequin 3.5.2.2.

|  | Rodent’s PAM | Human’s PAM | Rodent’s ENC-SOL | Human’s ENC-SOL |
| --- | --- | --- | --- | --- |
| 1F8A/15J15 | 0.67253 +- 0.01735 | 0.90518 +- 0.00779 | 0.00880 +- 0.00288* | 0.31672 +- 0.01512 |
| 1F8A/29E6A | 0.05767 +- 0.00761* | 0.00196 +- 0.00196* | 0.00000 +- 0.00000* | 0.34409 +- 0.01719 |
| 15J15 /29E6A | 0.00000 +- 0.00000* | 0.87097 +- 0.01042 | 0.03715 +- 0.00539* | 0.55230 +- 0.01452 |
| 1F8A/SM13-478 | 0.06256 +- 0.00658 | 0.00391 +- 0.00185* | 0.00293 +- 0.00164* | 0.16031 +- 0.01072 |
| 15J15/SM13-478 | 0.00000 +- 0.00000* | 0.15738 +- 0.01065 | 0.00391 +- 0.00233* | 0.21017 +- 0.01241 |
| 29E6A/SM13-478 | 0.00293 +- 0.00164* | 0.41251 +- 0.01353 | 0.15445 +- 0.01298 | 0.82991 +- 0.01156 |
| 1F8A/SMMS3 | 0.00782 +- 0.00280* | 0.38905 +- 0.01265 | 0.08602 +- 0.00784 | 0.21896 +- 0.01206 |
| 15J15/SMMS3 | 0.00978 +- 0.00294* | 0.29717 +- 0.01252 | 0.18671 +- 0.01193 | 0.29814 +- 0.01461 |
| 29E6A/SMMS3 | 0.00489 +- 0.00203* | 0.54545 +- 0.01571 | 0.02248 +- 0.00596* | 1.00000 +- 0.00000 |
| SM13-478/SMMS3 | 0.00684 +- 0.00336* | 0.04106 +- 0.00621* | 0.01173 +- 0.00363* | 0.03715 +- 0.00501* |
| 1F8A/SMMS16 | 0.26393 +- 0.01495 | 0.44184 +- 0.01532 | 0.08504 +- 0.00722 | 0.38319 +- 0.01423 |
| 15J15/SMMS16 | 0.00000 +- 0.00000* | 0.75855 +- 0.01351 | 0.78886 +- 0.01221 | 0.00000 +- 0.00000* |
| 29E6A/SMMS16 | 0.00098 +- 0.00098* | 0.14565 +- 0.01196 | 0.57478 +- 0.01724 | 0.54741 +- 0.01650 |
| SM13-478/SMMS16 | 0.00587 +- 0.00260* | 0.04497 +- 0.00523 | 0.71359 +- 0.01248 | 0.01760 +- 0.00522* |
| SMMS3/SMMS16 | 0.00098 +- 0.00098* | 0.34506 +- 0.01165 | 0.00098 +- 0.00098* | 0.36559 +- 0.01391 |
| 1F8A/SMMS18 | 0.84751 +- 0.01188 | 0.79374 +- 0.01162 | 0.65689 +- 0.01299 | 0.19550 +- 0.01166 |
| 15J15/SMMS18 | 0.03226 +- 0.00370* | 0.26588 +- 0.01607 | 0.01075 +- 0.00331* | 0.11535 +- 0.01008 |
| 29E6A/SMMS18 | 0.28543 +- 0.01506 | 0.41251 +- 0.01367 | 0.36070 +- 0.01298 | 0.73021 +- 0.01379 |
| SM13-478/SMMS18 | 0.07136 +- 0.00861 | 0.54154 +- 0.01309 | 0.00098 +- 0.00098* | 0.73118 +- 0.01190 |
| SMMS3/SMMS18 | 0.52102 +- 0.01450 | 0.10850 +- 0.01030 | 0.06647 +- 0.00812 | 0.20332 +- 0.01349 |
| SMMS16/SMMS18 | 0.58260 +- 0.01353 | 0.74291 +- 0.01208 | 0.12121 +- 0.00900 | 0.56012 +- 0.01732 |

* Significant results (*p* < 0.05)
