# Supplementary material for: Genetic analysis of Schistosoma mansoni in a low-transmission area in Brazil suggests population sharing between wild-hosts and humans and geographical isolation
Source: PLoS Negl Trop Dis. 2025 Aug 11;19(8):e0013379. doi: 10.1371/journal.pntd.0013379 (PMC12338815; doi:10.1371/journal.pntd.0013379)
Supplement: S5 Table — (DOCX) [file pntd.0013379.s007.docx]

**S5 Table:** Results for Hardy-Weinberg Equilibrium (HWE) Test evaluated with GenAIEx version 6.503 using Chi-squared test.

| Pop | Locus | DF | *χ^2^* | *p* | Signif |
| --- | --- | --- | --- | --- | --- |
| Rodent’s PAM | 1F8A | 15 | 12,524 | 0,639 | ns |
|  | 15J15A | 36 | 68,021 | 0,001 | *** |
|  | 29E6A | 10 | 51,065 | 0,000 | *** |
|  | SM13-478 | 15 | 41,008 | 0,000 | *** |
|  | SMMS 3 | 15 | 27,668 | 0,024 | * |
|  | SMMS 16 | 10 | 91,983 | 0,000 | *** |
|  | SMMS 18 | 3 | 3,297 | 0,348 | ns |
| Human’s PAM | 1F8A | 21 | 54,075 | 0,000 | *** |
|  | 15J15A | 15 | 43,739 | 0,000 | *** |
|  | 29E6A | 10 | 54,545 | 0,000 | *** |
|  | SM13-478 | 10 | 28,496 | 0,002 | ** |
|  | SMMS 3 | 10 | 22,951 | 0,011 | * |
|  | SMMS 16 | 6 | 22,253 | 0,001 | ** |
|  | SMMS 18 | 10 | 8,415 | 0,588 | ns |
| Rodent’s ENC-SOL | 1F8A | 28 | 48,315 | 0,010 | ** |
|  | 15J15A | 36 | 44,026 | 0,168 | ns |
|  | 29E6A | 28 | 239,111 | 0,000 | *** |
|  | SM13-478 | 78 | 305,008 | 0,000 | *** |
|  | SMMS 3 | 66 | 163,246 | 0,000 | *** |
|  | SMMS 16 | 36 | 254,756 | 0,000 | *** |
|  | SMMS 18 | 36 | 52,882 | 0,034 | * |
| Human’s ENC-SOL | 1F8A | 15 | 11,755 | 0,697 | ns |
|  | 15J15A | 6 | 14,000 | 0,030 | * |
|  | 29E6A | 3 | 0,426 | 0,935 | ns |
|  | SM13-478 | 21 | 19,778 | 0,535 | ns |
|  | SMMS 3 | 3 | 8,222 | 0,042 | * |
|  | SMMS 16 | 10 | 16,030 | 0,099 | ns |
|  | SMMS 18 | 3 | 1,184 | 0,757 | ns |

ns: not significant; **p* < 0.05; ***p* < 0.01; ****p <* 0.001
